# A phase II study of Epirubicin in oxaliplatin-resistant patients with metastatic colorectal cancer and *TOP2A* gene amplification

**DOI:** 10.1186/s12885-016-2124-5

**Published:** 2016-02-11

**Authors:** Line S. Tarpgaard, Camilla Qvortrup, Sune B. Nygård, Signe L. Nielsen, Diana R. Andersen, Niels Frank Jensen, Jan Stenvang, Sönke Detlefsen, Nils Brünner, Per Pfeiffer

**Affiliations:** Department of Oncology, Odense University Hospital, Odense, Denmark; Faculty of Health and Medical Sciences, Department of Veterinary Disease Biology, University of Copenhagen, Frederiksberg, Denmark; Department of Pathology, Rigshospitalet, University of Copenhagen, Copenhagen, Denmark; Department of Pathology, Odense University Hospital, Odense, Denmark

## Abstract

**ᅟ:**

The overall purpose of this study is to provide proof of concept for introducing the anthracycline epirubicin as an effective, biomarker-guided treatment for metastatic colorectal cancer (mCRC) patients who are refractory to treatment with oxaliplatin-based chemotherapy and have *TOP2A* gene amplification in their tumor cells.

**Background:**

Epirubicin is an anthracycline that targets DNA topoisomerase 2-α enzyme encoded by the *TOP2A* gene. It is used for treatment of several malignancies, but currently not in CRC. *TOP2A* gene amplifications predict improved efficacy of epirubicin in patients with breast cancer and thus could be an alternative option for patients with CRC and amplified *TOP2A* gene. We have previously analysed the frequency of *TOP2A* gene aberrations in CRC and found that 46.6 % of these tumors had *TOP2A* copy gain and 2.0 % had loss of *TOP2A* when compared to adjacent normal tissue. The *TOP2A* gene is located on chromosome 17 and when the *TOP2A*/CEN-17 ratio was applied to identify tumors with gene loss or amplifications, 10.5 % had a ratio ≥ 1.5 consistent with gene amplification and 2.6 % had a ratio ≤ 0.8 suggesting gene deletions. Based on these observations and the knowledge gained from treatment of breast cancer patients, we have initiated a prospective clinical, phase II protocol using epirubicin (90 mg/m2 iv q 3 weeks) in mCRC patients, who are refractory to treatment with oxaliplatin.

**Methods/Design:**

The study is an open label, single arm, phase II study, investigating the efficacy of epirubicin in patients with oxaliplatin refractory mCRC and with a cancer cell *TOP2A*/CEN-17 ratio ≥ 1.5. *TOP2A* gene amplification measured by fluorescence in situ hybridization. A total of 25 evaluable patients (15 + 10 in two steps) will be included (Simon’s two-stage minimax design). Every nine weeks, response is measured by computed tomography imaging and evaluated according to RECIST 1.1. The primary end-point of the study is progression-free survival.

**Trial registration:**

Eudract no. 2013-001648-79.

## Background

Colorectal cancer (CRC) is a major health problem since it is among the five most prevalent cancers [1, 2]. Over the last decade, the use of novel treatment modalities and complex treatment strategies in terms of optimized surgery, adjuvant chemotherapy, and novel targeted biological agents have contributed to significant improvement of outcome of the entire population of patients with CRC. However, only three cytotoxic drugs (fluorouracil (5-FU), oxaliplatin and irinotecan) are recommended as standard treatment in patients with metastatic CRC (mCRC). In Europe, the routine clinical management of patients with primary CRC involves adjuvant treatment with 5-FU/Leucovorin (LV) and oxaliplatin (FOLFOX, FLOX or XELOX) given to high-risk patients [3–6]. In the treatment of mCRC, patients who have yet not received oxaliplatin will often be offered FOLFOX, FLOX or XELOX, followed by 5-FU/LV and irinotecan (FOLFIRI, FLIRI or XELIRI) at progression, or vice versa [7–10]. Chemotherapy may be combined with the EGF-receptor targeting antibodies cetuximab or panitumumab in patients with *RAS* wild type tumors [11–14], while patients with *RAS* mutated tumors often will receive the VEGF targeting antibody bevacizumab [15, 16]. The objective response rate to first-line systemic treatment of mCRC patients approximates 50 % [10, 17]. However, only 10 % of mCRC patients will obtain objective response during second-line treatment [10], suggesting that there is a high degree of cross-resistance between the used drugs. Improvements in mCRC treatment may be achieved through the development of novel drugs with new molecular mechanisms of action and thus lack of cross-resistance with currently used drugs. However, drug development is expensive (estimated cost is 1 Bill USD per drug), takes a long time (12–15 years) and the risk of failure in late stage clinical trials is extremely high. An alternative to the drug development approach is to search for novel predictive biomarkers that can guide personalized treatment and thereby select the right drug for the right patient at the right time. Using predictive biomarkers will increase the therapeutic index and avoid side effects among the many patients who will not benefit from the treatment. A third way to address these medical problems is to test whether drugs being used in some other cancer types might have beneficial effects also in CRC. However, with an expected relatively low response rate, such drugs should only be used together with companion diagnostics allowing for a pre-treatment selection of patients with the highest likelihood of obtaining benefit from the treatment. This third approach is called biomarker guided repurposing and is the foundation for the present protocol, where we investigate whether epirubicin has a beneficial effect in oxaliplatin resistant mCRC patients with *TOP2A* gene amplification.

### Epirubicin and TOP2A

The anthracycline epirubicin exerts its antitumor effects by interference with the synthesis and function of DNA and is most active in the S-phase of the cell cycle [18]. It is metabolized in the liver and primarily eliminated in the bile. The main molecular target of epirubicin is the DNA topoisomerase 2-α enzyme (Top2α) that plays a key role in maintaining the topological status of chromosomes during DNA replication and transcription. During DNA transcription, Top2α removes DNA supercoiling, and at the end of DNA replication, Top2α is essential for chromosome condensation and segregation. In this process, Top2α reversibly binds and cleaves both complementary DNA strands forming what is called a Top2 cleaving complex (Top2cc). Epirubicin leads to entrapment of Top2α in the Top2CC and thereby prevents religation of the cleaved DNA strands, which ultimately leads to DNA damage. Apart from this, epirubicin also interferes with a broad range of DNA processes through DNA intercalation [19].

*TOP2A* amplification predicts improved efficacy to epirubicin in patients with breast cancer [20, 21] and thus could be an alternative option to irinotecan-based therapy in patients with CRC and *TOP2A* amplification who relapsed on oxaliplatin containing chemotherapy. We have previously established irinotecan (SN-38, the active metabolite of irinotecan) resistant CRC cell lines, and these CRC cells were also resistant to epirubicin [22]. In contrast, our SN-38 resistant CRC cells were also resistant to epirubicin whereas our oxaliplatin resistant CRC cell lines retained sensitivity to epirubicin [Niel Frank Jensen et al. Unpublished observations]. In another study we analysed the frequency of *TOP2A* aberrations in CRC tissue from 153 primary stage III CRC tumors and found that 46.6 % had *TOP2A* copy gain and 2.0 % *TOP2A* loss*,* when compared to adjacent normal tissue [23]. When the *TOP2A*/CEN-17 ratio was applied to identify tumors with gene loss or amplifications, 16 (10.5 %) had a ratio ≥ 1.5, consistent with gene amplification and 4 (2.6 %) had a ratio ≤ 0.8, suggesting gene deletion.

## Design/methods

### Design

The study is an open label, single arm, phase II study, investigating the efficacy of epirubicin in patients with oxaliplatin refractory mCRC and a cancer cell *TOP2A*/CEN-17 ratio ≥ 1.5. A second aim is the collection of relevant tumor and blood material for subsequent biomarker studies.

### Inclusion criteria

To be eligible for inclusion, patients must provide written informed consent. All patients must be above the age of 18 years, have WHO performance status 0–2, and a life expectancy of at least 3 months. Furthermore, patients must have histologically verified, non-resectable, oxaliplatin resistant mCRC i.e. progression during or after oxaliplatin-based therapy (at least 2 months of oxaliplatin-based palliative therapy or at least 4 months of adjuvant oxaliplatin-based therapy). Moreover, FFPE tumor tissue blocks (primary tumor biopsy/resection specimen or biopsy/resection specimen from a metastatic lesion) must be available for fluorescence *in situ* hybridization (FISH) analysis of the *TOP2A*/CEN-17 ratio and this ratio has to be ≥ 1.5.

### Treatment

Drug/dosage: Epirubicin 90 mg/m^2^ day 1 administration via fast running infusion of 0.9 % sodium chloride every 21-day. Treatment will continue until maximum cumulative dose of Epirubicin = 900 mg/m^2^, unacceptable toxicity, progressive disease at computer tomography (CT) scan according to RECIST version 1.1 or patients wish of ending treatment. Before start of treatment a baseline CT scan will be performed, and a new CT scan will be performed after every third series of epirubicin to monitor treatment response.

### Ethics

The study will be conducted in compliance with the protocol and in accordance with the ethical principles put forward in the second Declaration of Helsinki and in accordance with good clinical practice (GCP) rules. The trial was approved by the Ethics Committee of Region Syddanmark (2013-001648-79/ S-20130042) and by the Danish Medical Authority (Eudract no. 2013-001648-79).

### Study objectives

The primary end-point of the study is progression-free survival (PFS), defined as time from the first infusion of epirubicin to the first documented disease progression, according to RECIST version 1.1. Secondary end-points include overall survival (OS), response rate (RR), toxicity, and validation of tissue inhibitor of metalloproteinase-1 (TIMP-1) measured in plasma and immunohistochemically in tumor tissue, as biomarker for anthracycline sensitivity/resistance [24]. Additionally, the relationship between *TOP2A* and *TOP1* gene amplifications will be studied [25].

### Statistics

The number of evaluable patients (the sample size) is based on Simon’s two stages Mini-max design [26]. This design ensures early study termination if there is insufficient effect. Patients will be evaluated with CT scans every 9 weeks. In randomized trials on second-line therapy, PFS is around 4 months. A tumor control rate less than 10 % after 4 months (at the time of the second evaluation CT scan) is not clinically relevant. Assuming a significance level at 0.05 (α = 0.05) and a power at 80 % (β = 0.20) it can be calculated, that 15 patients should be included in the first part of the study. The enrolment will continue until 15 patients have completed the second CT scan or experienced progressive disease. If none out of the first 15 consecutive patients achieve stable disease at the second CT scan (i.e. after 6 courses of epirubicin), we will reject our hypotheses and close the study after the first stage of accrual. If one or more patients achieve tumor control (partial response or stable disease) at the second scan, an additional 10 patients will be accrued in the second stage. If 5 out of 25 patients achieve tumor control after six courses of therapy, a tumor control rate of 30 % cannot be rejected, and it will be concluded that the treatment is effective enough to continue with future studies.

We will use non-parametric methods for calculation of patient characteristics, side effects and disease control. PFS and OS will be calculated and reported as median survival (Kaplan-Meier method).

## Methods

The *TOP2A* FISH pharmDx™ Kit (Dako, Glostrup, Denmark) will be used according to the manufacturer’s instructions, as previously reported [23]. For every patient tumor sample, *TOP2A* and CEN-17 signals will be counted in 60 non-overlapping malignant cells, and a *TOP2A*/CEN-17 ratio will be calculated as the total counted number of *TOP2A* signals divided by the total counted number of CEN-17 signals. *TOP2A* FISH analysis will be performed on archived FFPE tumor tissues obtained from either the primary tumor (at time of diagnosis or obtained from the surgical resection specimen) or from a metastatic lesion (biopsy or resection specimen).

## Discussion

The number of treatment options in patients with mCRC is still very limited. Response rates decrease dramatically with time, while the patients proceed in the treatment lines. New and non-cross resistant treatment options are urgently needed, and biomarkers predictive of response to chemotherapeutic treatments are totally lacking. Studies on breast cancer patients indicate that amplification and possibly deletion of *TOP2A* is predictive of response to epirubicin [20, 21]. Data on *TOP2A* aberrations in CRC are sparse, with reported *TOP2A* amplification rates ranging from 2.2 to 46.6 % in studies using different analytical methods [23, 27, 28]. In a study of the frequency of *TOP2A* gene aberrations in CRC tissue, we found that a total of 10.5 % of the patients had a *TOP2A*/CEN-17 ratio ≥ 1.5, which is compatible with *TOP2A* amplification [23]. Previous studies have investigated the effect of epirubicin in the entire unselected group of patients with mCRC, but found that this treatment had lower impact than oxaliplatin- or irinotecan-based treatment options [18, 29–31]. Taken together, those data led to the hypothesis that re-purposing epirubicin to patients with mCRC and *TOP2A* gene amplification may represent a valid treatment offer to a subset of mCRC patients. Hence, the aim of this clinical study is to take a further step towards personalized mCRC treatment. If epirubicin should prove to be effective in patients with increased *TOP2A* gene copy numbers, this may lead to a novel personalized treatment option for patients with mCRC.

This study differs from the standard treatment strategy for mCRC, where the patients often will be offered irinotecan-based therapy after failure to oxaliplatin-based therapy or vice versa. Median PFS of this standard treatment is approximately 2 months, and 75 % of the patients will experience progressive disease after 4 months of treatment [10]. In the present phase II protocol with pre-selected patients, we expect that 30 % will have objective response after the first 4 months of treatment, thereby achieving an increased median PFS. All patients will be evaluated every 9 weeks, enabling a quick change in treatment strategy if a patient experiences disease progression during epirubicin treatment [32, 33]. Carefully planned and closely monitored “window of opportunity” phase II studies are feasible and ethically acceptable in mCRC patients, and may have the advantage to determine potential efficacy of novel agents without placing the patients at risk. In the present study, close monitoring of efficacy every 9 weeks will ensure that this novel treatment line with epirubicin will be offered without further delay in case of progression during epirubicin treatment, with respect to any future treatment option.

The statistical basis for the protocol is based on the fact that we expect a response rate of 30 % in this sub-population of patients with *TOP2A* aberrations, which is significantly better than the typical second line treatment these patients will be offered, which usually has response rates of 10 %. In conclusion, if the objectives of this study protocol are met, the expected 10 % of mCRC patients with a TOP2A/CEN-17 ratio ≥ 1.5 will be offered an extra line of effective treatment, which may lead to an overall improved survival in this subgroup of mCRC patients.

## Trial status

A total of 120 patients have been screened for *TOP2A* levels and we have found 14 patients (12 %) to have a *TOP2A*/CEN-17 ratio ≥ 1.5 in primary tumor, a metastatic lesion or both. To date 3 patients have been included and initiated treatment with epirubicin.
